# Clinico-pathological significance of exosome marker CD63 expression on cancer cells and stromal cells in gastric cancer

**DOI:** 10.1371/journal.pone.0202956

**Published:** 2018-09-17

**Authors:** Yuichiro Miki, Masakazu Yashiro, Tomohisa Okuno, Kenji Kuroda, Shingo Togano, Kosei Hirakawa, Masaichi Ohira

**Affiliations:** 1 Department of Surgical Oncology, Osaka City University Graduate School of Medicine, Osaka, Japan; 2 Molecular Oncology and Therapeutics, Osaka City University Graduate School of Medicine, Osaka, Japan; University of South Alabama Mitchell Cancer Institute, UNITED STATES

## Abstract

**Background:**

It has been reported that CD63, an exosome marker, is expressed in solid cancer tissues. However, its significance in patients with gastric cancer has not been clarified. Exosomes derived from cancer cells and stromal cells might play an important role in the intracellular communications involved in the development of carcinoma. This study aimed to clarify the relationship between CD63 expression in cancer cells and stromal cells and clinical-pathologic factors.

**Methods:**

A total of 595 gastric cancer patients were enrolled in this study. CD63 expression in cancer cells and stromal cells was analyzed by immunohistochemistry. The correlations between CD63 expression and several clinicopathological factors were investigated.

**Results:**

CD63 expression was mainly observed on the cell membranes of cancer cells, and in the cytoplasm of stromal cells. Of 595 patients, 247 cases had CD63-positive cancer cells, and 107 cases had CD63-positive stromal cells. Cases with CD63-positive cancer cells were significantly correlated with scirrhous-type gastric cancer, tumor depth, lymph node metastasis, lymphatic invasion, and tumor size. Cases with CD63-positive stromal cells were significantly correlated with age (≥65), tumor depth (T3-4), lymphatic invasion, and tumor size (≥ 5 cm). The 5-year survival rate was significantly lower (p<0.001) in patients with CD63-positive than CD63-negative tumors. Multivariate analysis showed that CD63 expression in cancer cells was a significant independent prognostic factor in patients with gastric cancer.

**Conclusion:**

CD63 might be a prognostic marker for patients with gastric cancer. CD63-positive exosomes might be associated with the interaction between stromal cells and cancer cells.

## Introduction

Recently, it has been reported that exosomes from cancer cells might be associated with intracellular communications involved in the development of the tumor microenvironment, such as metastatic niche formation and angiogenesis, resulting in the progression of carcinoma[1–4].

Exosomes are small membrane vesicles (30–150 nm) containing functional molecules, and they can be horizontally transferred to the surrounding cells[5]. Exosomal membranes are enriched with endosome-specific tetraspanins such as CD9, CD63, and CD81. Among these tetraspanins, in this study, we focused on CD63. There have been several articles regarding the association between CD63 and cancer. In pancreatic cancer, the expression of CD63 has been reported to be higher in cancerous tissues than in normal tissues[6]. Duijevesz *et al*. have reported that urine samples from prostate cancer patients included CD63-positive exosomes[7]. In addition, CD63 expression has been reported to be a prognostic factor in patients with gastrointestinal stromal tumor[8]. However, the significance of CD63 in patients with gastric cancer has not been fully investigated. Thus, the object of this study was to clarify the clinical significance of CD63 expression in cancer cells and stromal cells in patients with gastric cancer.

## Methods

### Patients

A total of 595 patients who had undergone a operation for the primary gastric cancer were enrolled in this study. We generated the tissue microarrays from these patients, and used for immunohistochemical staining. We made the pathologic diagnoses and classifications according to the UICC TNM classification of malignant tumors 7^th^ edition[9]. The study protocol followed the ethical guidelines of the Declaration of Helsinki. This study was approved by the Osaka City University ethics committee. We obtained written informed consent from all patients.

### Immunohistochemical determination of CD63

Immunohistochemical staining was analyzed using 595 gastric cancers. Slides were deparaffinized and rehydrated, and then heated for 10 min at 105°C in an autoclave in Target Retrieval Solution (Dako, Carpinteria, CA). After blocking endogenous peroxidase activity, we incubated the specimens with CD63 antibody (1:200; Life technologies) for 1 h at room temperature, and were incubated with biotinylated goat anti-rabbit IgG for 10 min. The slides were treated with streptavidin-peroxidase reagent, followed by counterstaining with Mayer’s hematoxylin. We evaluated CD63 expression at the invading tumor front. CD63 expression was evaluated by intensity of staining and percentage of stained cancer cells and stromal cells respectively: intensity was given scores 0–3 (0 = no, 1 = weak, 2 = moderate, 3 = intense), and the percentage of immunopositive cells was given scores 0–3 (0 = 0%, 1 = 10%, 2 = 20–30%, 3 = 40%-100%). The two scores were multiplied to obtain the decisive result of 0–9. Expressions were considered positive in tumor cells when scores were 2 or more and negative when scores were 0–1. Evaluation was made by two double-blinded independent observers who were unaware of clinical data and outcome.

### Statistical analysis

Associations between CD63 expression and clinicopathological findings were analyzed using the chi-square test. Overall survival (OS) was defined as the time from operation to death from any cause. OS curves were estimated by the Kaplan-Meier method and compared using the log-rank test. Multivariate analysis was carried out using cox proportional hazard model. All statistical analyses were performed using the JMP statistical software (version 8.0; SAS Institute, Cary, NC). Two-sided probability (p) value of < 0.05 was considered to be statistically significant.

## Results

### Relationship between CD63 expression and clinicopathological factors in gastric cancer

**Fig 1:**
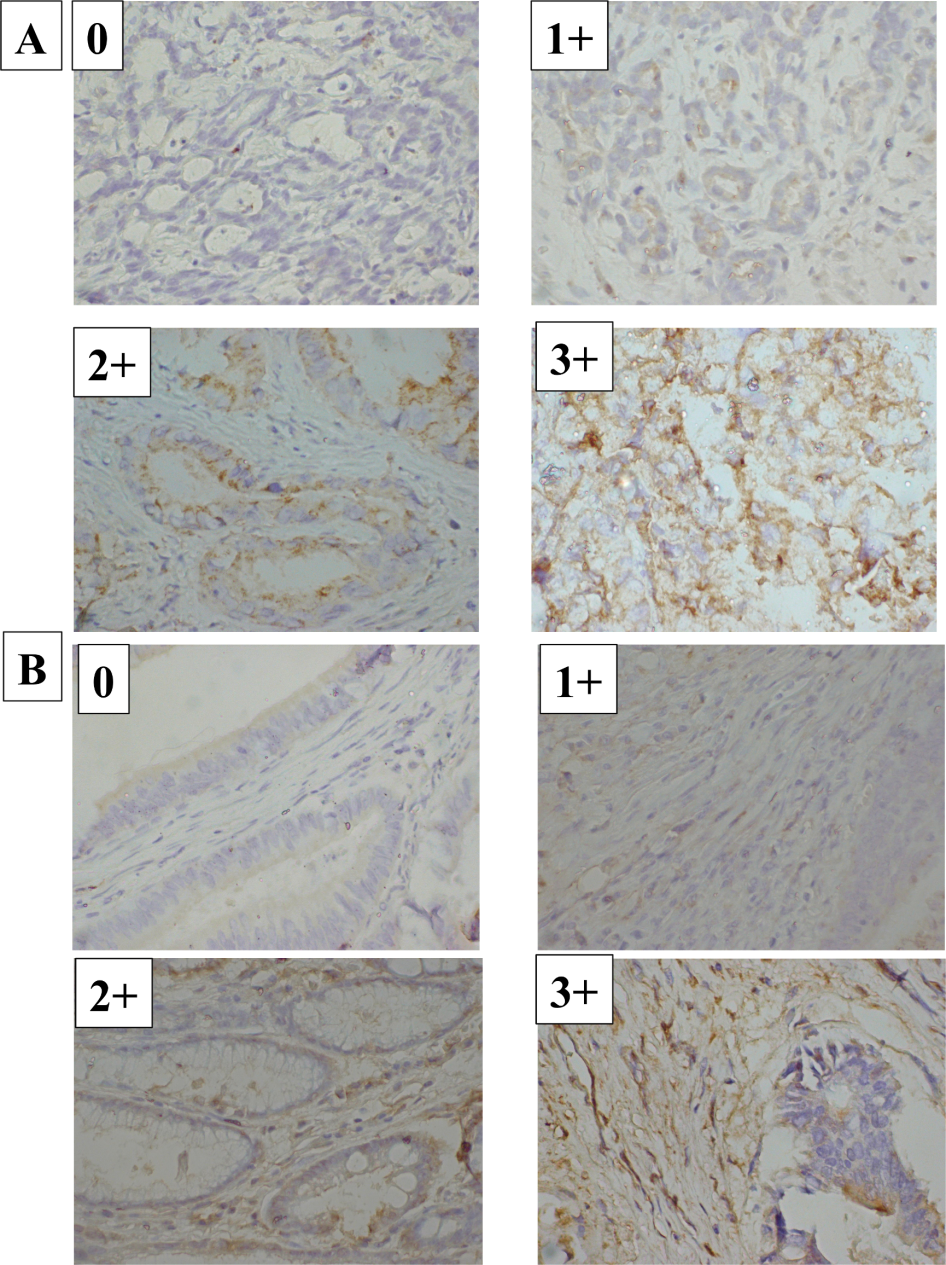
CD63 expression depending on the intensity of the staining. **(A)** Representative pictures of CD63 expression on cancer cells depending on the intensity of the staining. CD63 was mainly expressed on the cell membrane of gastric cancer cells, with a smaller amount in the cytoplasm. Pictures of score 0 and 1 are from patients with early gastric cancer, and both are well differentiated tubular adenocarcinoma. Picture of score 2+ and 3+ are from patients with advanced gastric cancer, and histologic type of score 2+ is well differentiated tubular adenocarcinoma, while histologic type of score 3+ is poorly differentiated adenocarcinoma. (B) Representative pictures of CD63 expression on stromal cells depending on the intensity of the staining. CD63 expression was mainly observed in the cytoplasm of stromal cells. Pictures of score 0 are from patients with early gastric cancer, and histologic type is well differentiated tubular adenocarcinoma. Pictures of score 1+ are from patients with advanced gastric cancer, and histologic type is poorly differentiated adenocarcinoma. Pictures of score 2+ are from patients with advanced gastric cancer, and histologic type is well differentiated tubular adenocarcinoma. Pictures of score 3+ are from patients with advanced gastric cancer, and histologic type is moderately differentiated tubular adenocarcinoma.

CD63 expression was mainly observed on the cell membranes of cancer cells, and in the cytoplasm of stromal cells (**Fig 1**). We evaluated the CD63 expressions of cancer cells and stromal cells in 595 gastric cancer tissues. Of 595 patients, 247 had CD63-positive cancer cells, and 107 had CD63-positive stromal cells. The correlation between CD63 expression and clinicopathological factors is shown in **Table 1.** The CD63 expression in stromal cells was significantly correlated with CD63 expression in cancer cells (p<0.0001). CD63 positivity in cancer cells was significantly correlated with scirrhous type (Type 4), tumor depth (T3-4), lymph node metastasis, lymphatic invasion, and tumor size (≥ 5 cm). CD63 positivity in stromal cells was significantly correlated with age (≥65), tumor depth (T3-4), lymphatic invasion, and tumor size (≥ 5 cm). The positivity of CD63 was higher in undifferentiated type than in differentiated type, but the difference between these two groups were not statistically significant.

**Table 1:** Correlation between CD63 expression and clinicopathologic factors in 595 patients with gastric cancer.

		CD63 expression on tumor cells		p value	CD63 expression on stromal cells		p value
		Negative (n = 348)	Positive (n = 247)		Negative (n = 489)	Positive (n = 107)	
Age	<65	162 (60.9%)	102 (39.1%)	0.20	226 (85.6%)	38 (14.4%)	0.040
	≥65	186 (56.2%)	144 (43.7%)		262 (79.2%)	69 (20.8%)	
Sex	Female	161 (60.8%)	103 (39.2%)	0.27	222 (84.1%)	42 (15.9%)	0.23
	Male	187 (56.3%)	144 (43.7%)		266 (80.4%)	65 (19.6%)	
Macroscopic type	0–3	327 (61.1%)	208 (38.9%)	<0.0001	443 (82.8%)	92 (17.2%)	0.15
	4	21 (35.0%)	39 (65.0%)		45 (75.0%)	15 (25.0%)	
Histologic type	Differentiated	179 (61.8%)	110 (38.1%)	0.097	229 (79.2%)	60 (20.8%)	0.086
	Undifferentiated	169 (55.2%)	137 (44.8%)		259 (84.6%)	47 (15.4%)	
Tumor depth	T1-2	240 (60.0%)	103 (30.0%)	<0.0001	295 (86.0%)	48 (14.0%)	0.0033
	T3-4	108 (42.8%)	144 (57.2%)		193 (76.6%)	59 (23.4%)	
Lymph node metastasis	Negative	225 (67.6%)	108 (32.4%)	<0.0001	280 (84.1%)	53 (15.9%)	0.048
	Positive	121 (46.5%)	139 (53.5%)		206 (79.2%)	54 (20.8%)	
Lymphatic invasion	Negative	191 (72.4%)	73 (27.6%)	<0.0001	226 (85.6%)	38 (14.4%)	0.048
	Positive	157 (47.6%)	173 (52.4%)		262 (79.4%)	68 (20.6%)	
Venous invasion	Negarive	292 (59.8%)	196 (40.2%)	0.15	403 (82.6%)	85 (17.4%)	0.44
	Positive	56 (52.3%)	51 (47.7%)		85 (79.4%)	22 (20.6%)	
Tumor size	< 5 cm	249 (69.0%)	112 (31.0%)	<0.0001	309 (85.6%)	52 (14.4%)	0.0060
	≥ 5 cm	98 (42.4%)	133 (57.6%)		177 (76.6%)	54 (23.4%)	
CD63 expression in stromal cells	Negative	337 (69.0%)	151 (31.0%)	<0.0001			
	Positive	11 (10.3%)	96 (89.7%)				

Values in parentheses are percentages unless indicated otherwise

### Survival

**Fig 2:**
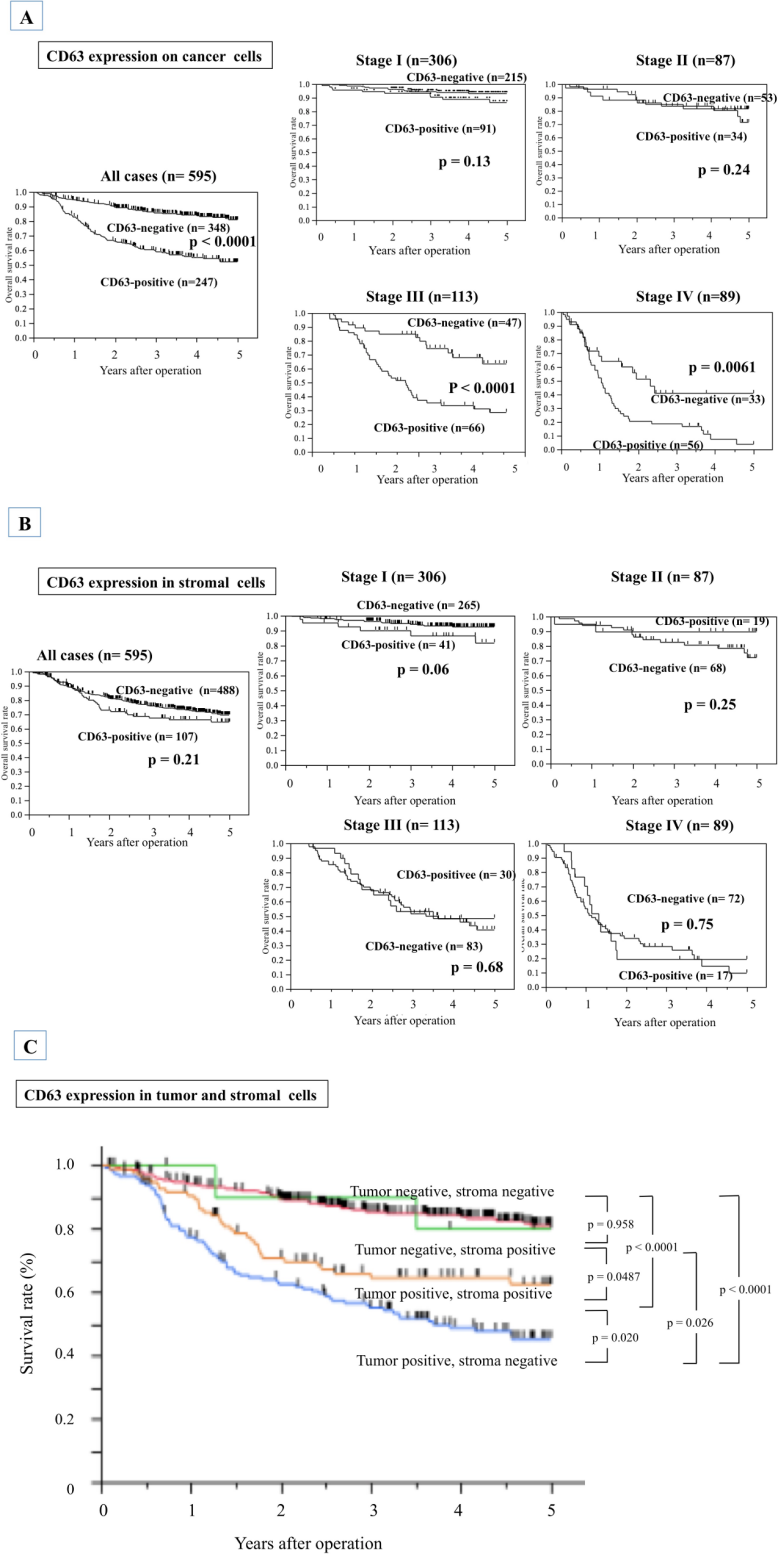
Survival of patients with gastric cancer. (A) Survival of gastric cancer patients with CD63 expression in cancer cells. The Kaplan–Meier survival curve indicates that the OS of patients with CD63-positive cancer cells was significantly worse than that of patients with CD63-negative expression (p<0.0001). Among patients with stage III and stage IV disease, the OS was significantly worse in those with CD63-positive cancer cells than those with CD63-negative expression. (B) The OS of patients with CD63-positive expression in stromal cells was not significantly different from that of patients with CD63-negative expression. (C) The OS of patients between four groups; both tumor and stroma positive, only tumor positive, only stroma positive, and both tumor and stroma negative.

The OS curves of patients by CD63 expression using the Kaplan-Meier method are shown in **Fig 2A**. The 5-year OS rates of patients with and without CD63 expression in cancer cells were 51.8% and 80.9%, respectively. Patients with CD63-positive cancer cells showed significantly worse OS than those with CD63-negative cancer (log–rank; p<0.0001). According to the analysis for each stage, the OS rates of stage III and stage IV patients with CD63 expression in cancer cells were significantly worse than those with CD63-negative expression (p < 0.0001 and p = 0.0063, respectively) (**Fig 2A)**. In contrast, the overall survival of patients with CD63-positive expression in stromal cells was not significantly different from that of patients with CD63-negative expression (p = 0.21). In the analysis by stage, the OS rate of stage I patients with CD63-positive stromal cells was worse than that of stage I patients with CD63-negative expression, although the difference was marginal (p = 0.06) (**Fig 2B)**. To investigate the effects on survival depending on CD63 positivity on tumor and stromal cells, we performed the survival analysis using 4 groups; CD63 positive both in tumor and stromal cells, CD63 postive only in tumor cells, CD63 positive only in stromal cells, and CD63 negative both in tumor and stromal cells. As a result patients with CD63 postive only in tumor cells showed the worst prognosis (log–rank; p<0.0001, Fig 2C).

Univariate and multivariate analyses using the proportional hazard model are shown in **Table 2**. Univariate analysis revealed that the overall survival of patients was significantly correlated with CD63 expression in cancer cells, age, macroscopic type-4 cancer, undifferentiated-type cancer, tumor depth (T3-4), lymph node metastasis, distant metastasis, venous invasion, and infiltration. Multivariable logistic regression analysis revealed that CD63 expression in cancer cells as well as age macroscopic type-4 cancer, undifferentiated-type cancer, tumor depth (T3-4), lymph node metastasis, distant metastasis, venous invasion and infiltration were independent predictive parameters for patient OS.

**Table 2:** Univariate and multivariate analyses with respect to overall survival after surgery in 597 patients with gastric cancer.

	Univariate		Multivariate	
	Odds ratio (95% CI)	P value	Odds ratio (95% CI)	P value
CD63 on tumor cells (Positive)	3.29 (2.38–4.60)	<0.0001	2.13 (1.51–3.02)	<0.0001
CD63 in stromal cells (Positive)	1.26 (0.85–1.81)	0.23	0.78 (0.51–1.15)	0.21
Age (≥ 65 y.o.)	1.52 (1.11–2.11)	0.009	1.43 (1.02–2.02)	0.038
Sex (Male)	1.24 (0.90–1.72)	0.18		
Macrosopic type (Type 4)	7.47 (5.25–10.5)	<0.001	2.68 (1.78–4.01)	<0.001
Histologic type (Undifferentiated)	1.94 (1.43–2.68)	<0.001	1.20 (0.84–1.73)	0.31
Tumor depth (T3 and T4)	6.56 (4.57–9.64)	<0.001	1.35 (0.80–2.33)	0.25
Lymph node metastasis (Positive)	8.38 (5.66–12.8)	<0.001	3.18 (1.89–5.52)	<0.001
Distant metastasis (Positive)	6.39 (3.59–10.5)	<0.001	2.51 (1.37–4.28)	0.0037
Lymphatic invasion (Positive)	5.53 (3.67–8.67)	<0.001	1.11 (0.66–1.94)	0.71
Venous invasion (Positive)	3.29 (2.36–4.53)	<0.001	1.67 (1.17–2.37)	0.0051
Tumor size (≥ 5 cm)	6.30 (4.45–9.10)	<0.001	1.40 (0.85–2.33)	0.19

## Discussion

In this study, we found that CD63 expression in gastric cancer cells was an independent significant prognostic factor. Because CD63 is a surface marker of exosomes, our data might suggest that exosomes derived from gastric cancer cells play an important role in cancer progression. To the best of our knowledge, this is the first study to investigate the clinico-pathological significance of exosome marker CD63 expression in cancer cells and stromal cells in gastric cancer.

CD63 expression was mainly observed on the cell membranes of cancer cells, and in the cytoplasm of stromal cells. Since CD63 expression in stromal cells was significantly correlated with CD63 expression on cancer cells, CD63-positive exosomes in the cytoplasm of stromal cells might be derived from the exosomes of cancer cells. These findings suggest that stromal cells might be affected by cancer cells via CD63-positive exosomes.

CD63 positivity in cancer cells was significantly correlated with scirrhous type (Type 4), tumor depth (T3-4), lymph node metastasis, lymphatic invasion, and tumor size (≥ 5 cm). These results might imply that gastric cancer cells, especially scirrhous-type cancer cells, secrete CD63-positive exosomes. On the other hand, CD63 positivity in stromal cells was significantly correlated with age (≥65), tumor depth (T3-4), lymphatic invasion, and tumor size (≥ 5 cm). Although the CD63-positive rate in stromal cells was less than that on cancer cells, patients with advanced cancer expressed CD63 in stromal cells as well as on cancer cells. These findings suggest that the interaction between stromal cells and cancer cells through CD63-positive exosomes might be active at the advanced stage.

Patients with CD63-positive cancer showed worse prognosis than those with CD63-negative cancer in this study. CD63 expression was an independent prognostic factor in this analysis, and thus might be a prognostic marker for patients with gastric cancer. In a previous report, CD63 was shown to play an important role in metastatic niche formation[10]. Grunwald *et al*. reported that CD63 is a receptor for the tissue inhibitor of metalloproteinase-1 (TIMP1), which creates a tumor niche in the liver microenvironment, resulting in liver metastasis of pancreas cancer. They demonstrated that formation of liver metastasis from injected pancreatic cancer cells was not observed in TIMP1 or CD63 knockout mice, and concluded that TIMP1 from pancreatic cancer activates hepatic stellate cells (HSCs) via its receptor CD63 and mediates niche formation for metastasis. In addition, co-expression of CD63 and TIMP1 have also been shown in patients with glioblastoma and astrocytoma by the other group[11]. Considering these data, CD63-positive exosomes of gastric cancer might also be associated with metastatic niche formation. In addition, CD63-positive exosomes from stromal cells might stimulate the malignant potential of cancer cells.

Among stage III and IV patients, CD63-positive cases showed significantly worse prognosis than CD63-negative cases. CD63-positive cases are highly metastatic and tend to relapse in the same stage. Cancer cells influence distant normal cells via exosomes[12], and CD63 plays a role in metastatic niche formation[10]. In the stage I and II patients, prognosis of was not significantly related to CD63 positivity and negativity. Circulating exosomes derived from cancer cells might be at very low levels in patients with early disease.

In conclusion, CD63 might be a prognostic marker for patients with gastric cancer. CD63-positive exosomes might be associated with the malignant potential of cancer cells through the interaction between stromal cells and cancer cells.

## Supporting information

S1 Table (XLSX)
